# Colorectal cancer survival in Greater Cuiabá, state of Mato Grosso, Brazil

**DOI:** 10.1590/1980-549720230022.2

**Published:** 2023-03-27

**Authors:** Gustavo Monteiro da Silva, Rita Adriana Gomes de Souza, Fernanda Cristina da Silva de Lima, Romero dos Santos Caló, Amanda Cristina de Souza Andrade, Bárbara da Silva Nalin de Souza, Flávio de Macêdo Evangelista, Noemi Dreyer Galvão

**Affiliations:** Universidade Federal de Mato Grosso, Instituto de Saúde Coletiva, Programa de Pós-Graduação em Saúde Coletiva – Cuiabá (MT), Brasil.; Instituto Nacional de Câncer – Rio de Janeiro (RJ), Brasil.; Universidade Federal de Mato Grosso, Instituto de Saúde Coletiva – Cuiabá (MT), Brasil.; Secretaria de Estado de Saúde de Mato Grosso – Cuiabá (MT), Brasil.

**Keywords:** Survival, Colorectal neoplasms, Demographic factors, Registries, Sobrevida, Neoplasias colorretais, Fatores demográficos, Sistema de registros

## Abstract

**Objective:**

To analyze the specific five-year survival of colorectal cancer (CRC) diagnosed between 2008 and 2013, according to sex and age group, of residents in Greater Cuiabá, state of Mato Grosso, Brazil.

**Methods:**

This is a retrospective cohort study. Specific survival of CRC was considered as the time between disease diagnosis and death from CRC, in months. Data from the Population-Based Cancer Registry and the Brazilian Mortality Information System were used. To estimate the probability of survival by sex and age group, the Kaplan-Meier estimator was used, and to estimate the effect of age group on the survival of participants, the Cox model stratified by sex was adjusted.

**Results:**

From 2008 to 2013, 683 new cases and 193 deaths from CRC were registered. The median time between diagnosis and death from CRC was 44.8 months (95%CI 42.4– 47.3) for women and 46.1 months (95%CI 43.4–48.6) for men, and the five-year survival probabilities of 83.5% (95%CI 79.9–87.2%) and 89.6% (95%CI 86.4–93.0%), respectively. Men aged 70–79 years (HR=2.97; 95%CI 1.11–3.87) and 80 years or older (HR=3.09; 95%CI 1.31–7.27) were at higher risk of mortality, and we verified no difference for women.

**Conclusion:**

Women had a shorter time between the diagnosis of CRC and death from the disease as well as a lower probability of survival. Conversely, men were at higher risk of mortality after 70 years of age.

## RESUMO

**Objetivo:**

Analisar a sobrevida específica em cinco anos do câncer colorretal diagnosticado entre 2008 e 2013, segundo sexo e faixa etária, de residentes na Grande Cuiabá, Mato Grosso.

**Métodos:**

Estudo de coorte retrospectiva. A sobrevida específica pelo câncer colorretal foi considerada como o tempo entre o diagnóstico da doença até o óbito por câncer colorretal, em meses. Utilizaram-se dados do Registro de Câncer de Base Populacional e do Sistema de Informações sobre Mortalidade. Para estimar a probabilidade de sobrevida por sexo e faixa etária, utilizou-se o estimador de Kaplan-Meier, e, para estimar o efeito da faixa etária na sobrevida dos participantes, foi ajustado modelo de Cox estratificado por sexo.

**Resultados:**

De 2008 a 2013, registraram-se 683 casos novos e 193 óbitos por câncer colorretal. O tempo mediano entre o diagnóstico e a morte por câncer colorretal foi de 44,8 meses (IC95% 42,4–47,3) para as mulheres e 46,1 meses (IC95% 43,4–48,6) para os homens e a probabilidade de sobrevida em cinco anos de 83,5% (IC95% 79,9–87,2%) e 89,6% (IC95% 86,4–93,0%), respectivamente. Os homens com 70-79 anos (HR=2,97; IC95% 1,11–3,87) e com 80 anos ou mais (HR=3,09; IC95% 1,31–7,27) apresentaram maior risco de mortalidade e sem diferença para as mulheres.

**Conclusão:**

O sexo feminino apresentou menor tempo entre o diagnóstico e o óbito pela doença, assim como menor probabilidade de sobrevida. Em contrapartida, foram os homens que apresentaram maior risco de mortalidade a partir dos 70 anos.

## INTRODUCTION

Chronic diseases and noncommunicable diseases account for more than half of the total number of deaths in Brazil, representing 54.7% of deaths registered in the country in 2019. Among the main groups of chronic diseases is cancer^1^, whose mortality has been growing worldwide and already represents the second leading cause of death in most countries^2^.

Cancer can affect several parts of the human body. Colorectal cancer (CRC) is a malignant neoplasm that affects the large intestine. Until 1950, it was relatively rare, but currently it is one of the cancers with the highest incidence and mortality worldwide^3^. This increase has been partly related to the aging of the population, but also to factors related to lifestyle such as unhealthy eating habits, smoking, sedentary lifestyle, alcohol consumption, and obesity^4,5^.

For 2020, more than 1.9 million new cases and 935 thousand deaths from CRC were estimated worldwide, representing about 1 in 10 cases or deaths from cancer. Overall, CRC ranks third in terms of incidence, but second in terms of mortality. Incidence rates are lower in middle-income countries, but mortality rates are higher^3^.

Concerning Brazil, for each year of the triennium 2020–2022, 20,520 cases of CRC are estimated in men and 20,470 in women. These values correspond to an age-adjusted incidence of 18.80 new cases/100 thousand men and 13.36 new cases/100 thousand women. For Greater Cuiabá, state of Mato Grosso, 20 cases of CRC were estimated for men and 40 for women in the year 2020, with an age-adjusted incidence of 8.58 and 14.04/100 thousand inhabitants for men and women, respectively^6^.

In terms of mortality, in 2019, 10,191 deaths from the disease were registered for men and 10,385 for women in Brazil, corresponding to a mortality rate of 9.73 deaths/100 thousand men and 7.81 deaths/100 thousand women, respectively^7^.

As for five-year net survival in the country, it was estimated at 44.5, 50.6, and 48.3% for patients diagnosed with colon cancer in the years 2000 to 2004, 2005 to 2009, and 2010 to 2014, respectively. For patients diagnosed with rectal cancer, for these same periods, the net survival was 37.7, 45.7, and 42.4%^8^.

Hospital-based studies have shown greater and better survival for women, young people, and patients diagnosed early^9–12^. The stage of CRC in diagnosis is one of the most important primary determinants of survival and one of the main predictors of mortality^13^. Five-year survival rates may be higher than 90% if the diagnosis is made at an early stage; however, only 37% of cases are diagnosed at this stage^14^. CRC can be considered a condition with good prognosis, and survival can be better the more initial the stage of the lesion at diagnosis is^8^.

Thus, the objective of this study is to analyze the specific five-year survival of CRC diagnosed between 2008 and 2013, according to sex and age group, of residents in Greater Cuiabá, state of Mato Grosso (MT), Brazil.

## METHODS

This is a population-based retrospective cohort study of residents of Greater Cuiabá. The municipalities of Cuiabá and Várzea Grande compose the region called Greater Cuiabá. They are the most populous municipalities in the state and have a territorial extension of 4,015,975 km^2^. According to data from the last demographic census (2010), Cuiabá recorded 4.5% of illiteracy rate, 20.0% of the population with income lower than half minimum wage, unemployment rate of people aged 16 years or older of 6.4%, and municipal human development index (MHDI) of 0.785. For Várzea Grande, these values were, respectively, 5.5; 27.3; 6.8; and 0.734%^15,16^.

Information on new cases of the disease were retrieved from the Population-based Cancer Registry of Greater Cuiabá, namely PBCR-Cuiabá, and information on deaths were provided by the State Department of Health of Mato Grosso (*Secretaria de Estado de Saúde de Mato Grosso* – SES-MT) and later comprised the Brazilian Mortality Information System (*Sistema de Informações sobre Mortalidade* – SIM). Those who presented the following codes were identified as cases or deaths due to CRC, registered according to the 10th edition of the International Classification of Diseases (ICD-10): C18 (malignant neoplasm of colon), C19 (malignant neoplasm of rectosigmoid junction), C20 (malignant neoplasm of rectum), and C21 (malignant neoplasm of anus and anal canal), as considered by the Brazilian National Cancer Institute (INCA)^6,17^. All new cases diagnosed with other malignant neoplasms were excluded from the analysis, as well as cases with diagnosis of tumors with in situ behavior; in situations in which the same patient had more than one primary tumor, only the first diagnosis was preserved.

The specific five-year survival of CRC was considered as the time, in months, elapsed between the diagnosis of the disease (time of patients’ entry in the cohort, whose recruitment occurred in the period from 01/01/2008 to 12/31/2013, i.e., diagnosed in this period) and death from CRC or date of last information^18^. The follow-up time occurred until 12/31/2018. The follow-up of new cases was passively performed, i.e., by cross-referencing the cases identified in the PBCR-Cuiabá with the deaths, regardless of the cause, identified in the SIM. Patients who were not found in death records (n=439; 64.3%) were assumed to be, in their vital status, alive and censored on 12/31/2018. Hence, there was no active follow-up of patients^19^. Patients who died from other types of cancer (n=21; 3.1%) and other causes (n=30; 4.4%) were assumed to be, in their vital status, as alive and censored on the date of death.

**Figure 1.**
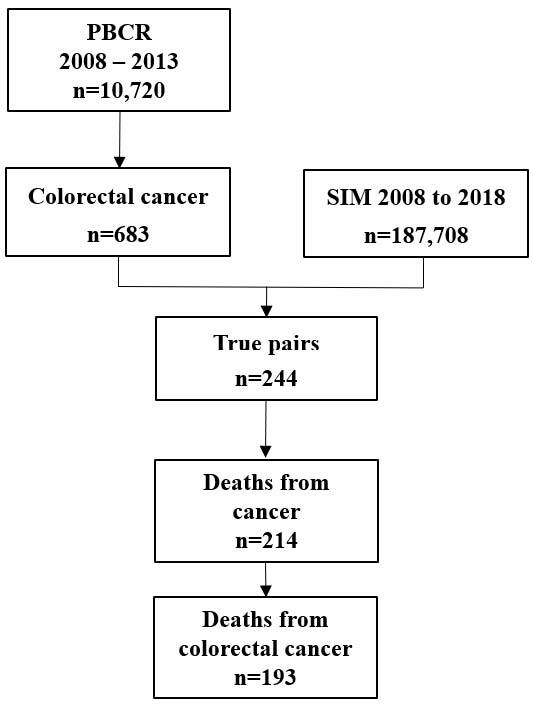
Flowchart of the probabilistic record linkage procedure between the Population-Based Cancer Registry of Greater Cuiabá and the Brazilian Mortality Information System databases.

PBCR: Population-based Cancer Registry; SIM: Brazilian Mortality Information System.

For the probabilistic linkage between the two databases (PBCR-Cuiabá and SIM), the record linkage technique was used, which aims to identify records related to the same unit (e.g., people) in two or more distinct databases^20^. To this end, three stages were performed: standardization of the common fields to be used in pairing; blocking, by the variable “sex”; and, finally, pairing, by developing concordance scores by the variables “patient’s name,” “mother’s name,” and “date of birth.” The soundex option was used, which reduces problems of spelling errors. A score was estimated for each pair of registries found. The higher the score, the greater the probability that the identified pair refers to the same person. A cutoff point of 7 was adopted, according to Queiroz et al.^21^. The databases were paired by the RecLink III program. Figure 1 shows the flowchart of the probabilistic record linkage procedure between PBCR-Cuiabá and SIM databases for the CRC, for the period from 2008 to 2013.

To estimate the probability of the specific five-year survival, by sex and age group, the Kaplan-Meier^19,22^ estimator and the log-rank test were used, aiming at verifying whether there were statistical differences in the lifetime per groups. To estimate the effect of the age group on the participants’ survival, stratified by sex, the Cox model was adjusted, obtaining estimates of the hazard ratio (HR) and their respective 95% confidence intervals (95%CI) for each category of interest. A statistical significance level of 0.05 was adopted. To verify the proportionality of the failure rates, the Schoenfeld residual test was used according to the statistical significance level of 5%^23^.

Statistical analyses were performed based on the results and graphs were obtained using the R software, version 4.0.2.

The project was approved by the Research Ethics Committee of Hospital Universitário Júlio Muller (Opinion No. 3.048.183, from 11/20/2018) and the Research Ethics Committee of Secretaria de Estado de Saúde de Mato Grosso (Opinion No. 3.263.744, from 04/12/2019).

## RESULTS

In the analyzed cohort, 683 individuals diagnosed with CRC were included. Of these, 244 died, 193 of which from CRC (Figure 1). Among deaths from CRC, 57.5% were women, and there was a higher proportion in the age groups from 60 to 69 years (31.5%) and 50 to 59 years (22.5%) for women and 70 to 79 years (29.3%) and 50 to 59 years (24.4%) for men. The time elapsed from diagnosis to death ranged from 38.7 months (70 to 79 years) to 49.1 months (under 50 years of age) for women; and from 33.2 months (80 years or older) to 47.5 months (50 to 59 years) for men. Women had a median of 44.8 months (95%CI 42.4–47.3) and men, 46.1 months (95%CI 43.4–48.6) (Table 1).

**Table 1.** Probability of specific survival of colorectal cancer and median time between diagnosis and death from colorectal cancer, according to sex and age group, Greater Cuiabá, state of Mato Grosso, Brazil, 2008 to 2013.

Sex	New cases of CRC (n=683*)	Deaths from CRC (n=193)	Five-year survival % (95%CI^†^)	Median time^‡^ % (95%CI)
Women (years)	374	111	83.5 (79.9–87.2)	44.8 (42.4–47.3)
<50	90	21	87.4 (81.1–94.3)	49.1 (47.0–51.2)
50 to 59	89	25	84.2 (57.2–88.4)	43.9 (41.4–46.3)
60 to 69	93	35	79.4 (72.0–87.7)	43.0 (40.6–45.5)
70 to 79	66	21	80.5 (71.7–90.6)	38.7 (36.0–41.4)
80 or older	33	9	81.3 (69.1–95.7)	39.8 (37.1–42.5)
Men (years)	309	82	89.6 (86.4–93.0)	46.1 (43.4–48.6)
<50	74	15	87.7 (80.6–95.4)	46.7 (44.1–49.3)
50 to 59	78	20	85.5 (78.4–93.2)	47.5 (45.2–49.8)
60 to 69	74	15	87.6 (80.5–95.3)	43.2 (40.4–46.0)
70 to 79	63	24	78.1 (68.9–88.7)	39.6 (36.6–42.6)
80 or older	18	8	69.9 (51.9–93.5)	33.2 (30.2–36.2)

CRC: colorectal cancer. *Ignored age: women=3; men=2; ^†^95%CI: 95% confidence interval; ^‡^In months.

The probability of specific five-year survival of CRC, considering both sexes, was 84.2% (95%CI 80.5–87.8%). For women, it was 83.5% (95%CI 79.9–87.2%), ranging from 79.4% (60 to 69 years) to 87.4% (under 50 years of age) and, for men, it ranged from 69.9% (80 years or older) to 87.7% (under 50 years of age), with survival of 89.6% (95%CI 86.4–93.0%) (Table 1).

We noticed that, for women, the lowest probabilities of survival occurred for age groups as from 60 years, especially for the age group from 60 to 69 years, which presented a value 10.3% lower than the same age group for men. Conversely, for men, the lowest probabilities of survival occurred as from 70 years, especially for those aged 80 years or older, who presented a value 16.3% lower than the same age group for women (Table 1).

**Figure 2.**
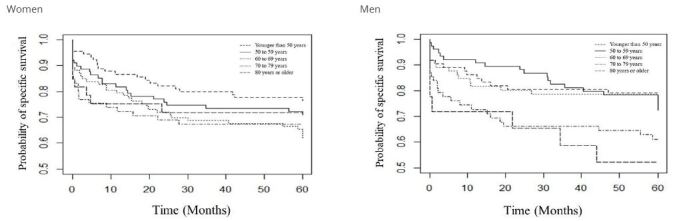
Specific five-year survival curve of colorectal cancer, according to sex and age group, Greater Cuiabá, state of Mato Grosso, Brazil, 2008 to 2013.

In Figure 2 we show the specific five-year survival curve of CRC, according to sex and age group. For women, we noticed that those aged 60 to 69 years and those aged 70 to 79 years had lower probabilities of survival when compared with women of other age groups, while those under 50 years of age had the highest probabilities. Moreover, we observed lower changes in probabilities as from 30 months.

For men, we observed the lowest probabilities of survival for the age groups 70 to 79 years and 80 years or older, while the other age groups presented similar probabilities after 47 months. There was a greater discrepancy between the curves, with values with greater amplitude between the age groups for this group (Figure 2).

In the Cox model, men aged 70 to 79 years (HR=2.97; 95%CI 1.11–3.87) and 80 years or older (HR=3.09; 95%CI 1.31–7.27) presented higher risk of mortality than men of other age groups. For women, there was no difference between age groups (Table 2).

**Table 2.** Hazard ratio of death from colorectal cancer and their respective 95% confidence intervals, according to sex and age group, Greater Cuiabá, state of Mato Grosso, Brazil 2008 to 2013.

Age group (years)	Women		Men	
	HR	95%CI	HR	95%CI
<50	1.00		1.00	
50 to 59	1.31	(0.75–2.30)	1.72	(0.91–3.23)
60 to 69	1.61	(0.94–2.75)	1.26	(0.64–2.49)
70 to 79	1.76	(0.98–3.15)	2.97	(1.11–3.87)
80 or older	1.54	(0.73–3.25)	3.09	(1.31–7.27)

HR: hazard ratio; 95%CI: 95% confidence intervals.

**Figure 3.**
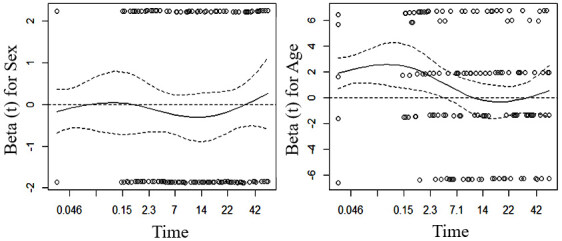
Assumption of proportional risks using standardized Schoenfeld residuals.

In Figure 3 we present the evaluation of the assumption of proportionality by Schoenfeld’s residual analysis. We can observed that there are no significant trends for the variables “sex” and “age.” It is noteworthy that the residuals do not have a random pattern around 0, thus suggesting a violation of the principle of proportionality of the risk function.

## DISCUSSION

The results of the present study showed that women had a shorter time between the diagnosis of CRC and death from the disease when compared with men as well as a lower probability of survival. For women, the lowest probabilities of survival were as from 60 years, while for men, as from 70 years. Conversely, men were at greater risk of mortality after 70 years of age.

Worldwide, the survival of the several types of cancer has been analyzed and published by the CONCORD program, using population-based registries. Brazil also contributes data to the program, from six national registries. However, the employed methodology estimates net survival, a survival function derived from excess risk, an estimator used in the comparison of populations because it is independent of population mortality^24^.

The latest data published by the program showed that for patients diagnosed with colon and rectal cancer from 2010 to 2014, five-year net survival has considerably varied, especially in Central and South America, Asia, and Europe. For this period, Brazil had a net survival of 48.3% for colon cancer and 42.4% for rectal cancer. The program highlights that most estimates were considered reliable, with the exception of some countries, including Brazil^8^.

A study that analyzed specific five-year survival of CRC, with data from 1980 to 2010 from major public hospitals in South Australia over three decades, showed that survival was 55.3% for men and 57.0% for women, but with no significant difference. Those aged 80 years or older had a lower probability of survival (49.0%) and a higher risk of death (HR=1.44; 95%CI 1.14–1.81)^25^.

CRC survival data are scarce in Brazil, especially specific data and with analysis per five-year period. In a study by Aguiar Junior et al.^26^, the authors did not analyze specific survival, but the overall survival (death from any cause), and did not consider anal canal cancer of patients with CRC treated in an oncology center in São Paulo (state of São Paulo), from 2000 to 2013, with follow-up until 2018. The results showed that five-year survival was 63.5%, with no difference between men and women, and higher in patients under 49 years (70.0%) and worse in those over 75 years (43.8%, p<0.001).

To date, only one study that evaluated survival in the same geographic region considered in the present study has been identified. In the study by Alves^27^, survival was analyzed considering patients diagnosed with CRC in the period from 2000 to 2009, and the follow-up period of five years. Nevertheless, anal canal cancer was not considered for the analyses. A total of 692 cases were analyzed, of which 347 were men and 344, women. The results of the study showed that specific survival was higher among women (62.8%; 95%CI 57–68%) than among men (57.2%; 95%CI 51.3–62.7%), and men had a 22% higher risk of death, but differences between the sexes were not significant.

Thus, we understand that the survival results in this study are, overall, higher than those previously presented. Information on the survival of cancer patients in a population allows comparing the efficacy of health systems, and the long-term surveillance of these results contributes to the evidence of the regional cancer control policy^28^.

Women in this study had lower survival than men, a result that is opposed to what has been verified in the literature^29,30^. Nonetheless, women have been screened for CRC at rates significantly lower than men^31^. Screening provides opportunities for early diagnosis of CRC; however, it often occurs when patients already present with signs and symptoms of the disease, a situation more frequent among men^32^, which can interfere with the probabilities of survival. In a meta-analysis study that verified the differences between sexes in the survival of CRC, the authors argue that this variable is rarely identified as an independent prognostic factor in clinical trials involving CRC patients, for example, as it has not been considered as a possible source of interaction between treatment and survival^33^.

Older men had a higher risk of death from CRC than younger men. Survival decreases with increasing age and this has been a predictive factor for death in cancer patients because it is more associated with a higher risk of comorbidities^9,34^.

For CRC, despite being a type of cancer whose signs and symptoms take years to appear, being susceptible to early detection or secondary prevention, late diagnosis leads to more invasive and expensive treatments and, consequently, lower survival for the patient and greater chance of mortality from the disease^35^.

It is worth highlighting that survival expresses the natural history of the disease, as well as cancer control activities, including screening, organization, and quality of health services^36^.

In Brazil, there is no population-based screening program for CRC. The Ministry of Health recommends that patients diagnosed or suspected of CRC or anal canal cancer should have preference in referral to the proctologist, and that the criteria should be readapted according to the needs of the local regulation. For those with family history or suspected Lynch Syndrome or familial adenomatous polyposis, screening should be done in a specialized genetics and gastroenterology service^37^.

Despite the discussions on the cost-benefit of implementing a national screening program for the disease and the organization of health services for this routine, considering the conditions of providing definitive diagnosis and treatment for the screened condition^38^, studies show a reduction in incidence and mortality from CRC with organized screening^39^.

As limitations of this study, we can mention that, usually, population-based survival does not enable the evaluation of variables such as tumor staging, morphology, and treatment, which are important for understanding the clinical feature of the disease^19^. Clinical and demographic variables, for not being mandatory, still have limited completion, which makes it difficult to monitor variations in survival data^40^. Moreover, limitations related to the passive follow-up of patients should be considered. Cases of patients who may have died, but which, due to failures in the death certificate (DC), were not registered and, consequently, not included in the analysis, may overestimate survival^19^. Another limitation is the lack of information on the life table of Greater Cuiabá, which made it impossible to estimate net survival^24^.

Conversely, the Kaplan-Meier method eliminates the need to assume that the censoring of observations uniformly occurs during this interval. In addition, the use of population-based data decreases the probability of selection bias because it includes all incident cases in the coverage region, which facilitates international comparations, as clinical trials include selected groups of patients^19^. The PBCR-Cuiabá was implemented in 1999 by the State Department of Health of Mato Grosso and currently has 38 health facilities as notifying sources, namely: one federal hospital, ten municipal and four state health services, six philanthropic establishments, and seventeen private health institutions (diagnostic and treatment clinics and anatomic pathology laboratories)^41^.

In 80% of countries, the increasing trend of premature cancer mortality is impacting the achievement of target 3.4 of the Sustainable Development Goals, which refers to reducing by one third premature mortality from chronic noncommunicable diseases by 2030^2^. Thus, the importance of the implementation, maintenance, updating, and availability of population data from Cancer Registries is evident, for the best knowledge of the disease panorama. The contributions would add to the structuring and formulation of public policies aimed at improving the early diagnosis, treatment, and quality of life of the population.
